# Assessment of choroidal vascularity and choriocapillaris blood perfusion in Chinese preschool-age anisometropic hyperopic amblyopia children

**DOI:** 10.3389/fped.2022.1056888

**Published:** 2022-11-17

**Authors:** Wang Hui, Hu Xiaofeng, Xin Hua, Dong Yihan, Tao Yong

**Affiliations:** Department of Ophthalmology, Beijing Chaoyang Hospital, The Third Clinical Medical College of Capital Medical University, Beijing, China

**Keywords:** hyperopia, amblyopia, choroidal vascularity index, anisometropic, choriocapillaris blood perfusion choroidal thickness

## Abstract

**Purpose:**

To determine the macular and peripapillary area choroid microstructure parameters of hyperopic anisometropic amblyopia eyes and compare to fellow and age-matched control eyes. To assess the correlation between the axial length (AL), choroidal thickness (CT) and choroid microstructure parameters.

**Methods:**

This cross-sectional comparative, non-interventional study involved 52 hyperopic anisometropic amblyopia children and 48 age-matched heathy controls. 52 eyes with hyperopic anisometropic amblyopia and 48 age-matched control eyes were studied. The peripapillary and subfoveal CT were determined. The total choroidal area (TCA), luminal area (LA), and stromal area (SA) of the subfoveal and peripapillary choroid were measured. In addition, the correlation between the AL, CT and choroid microstructure parameters were calculated.

**Results:**

The peripapillary and subfoveal CT of the amblyopic eyes was significantly thicker than the fellow and control eyes (all *P* < 0.05). The subfoveal and peripapillary choroidal SA, LA and TCA of the amblyopic eyes were significantly increased than that of the fellow and control eyes (all *P* < 0.05). The choroidal vascularity index (CVI) values of the amblyopic eye were significantly different among the three groups (*P* < 0.05). There was a statistically significant negative correlation between AL and subfoveal CT (SFCT), LA and TCA levels (*P* < 0.001, *P* = 0.039, *P* = 0.027, respectively). Spherical equivalent (SE) was positive correlated with SFCT, LA and TCA levels (*P* = 0.456, 0.229 and 0.240, respectively; all *P* < 0.05). There was a statistically significant positive correlation between SFCT, SE, LA, SA, TCA and CVI levels (all *P* < 0.05).

**Conclusion:**

The subfoveal and peripapillary CT of amblyopic children abnormally increased and correlated with shorter AL and higher SE. The choroidal structure of the amblyopic eyes was different from the fellow and control eyes, the hyperopic anisometropic amblyopic eyes had significantly thicker sub-foveal choroid, higher LA, SA, and TCA. AL and CT affect choroidal structure and vascular density. Choroidal blood flow may be increased in amblyopic eyes. The larger LA, SA, TCA, and lower CVI were characteristic of the amblyopic eye.

## Introduction

Amblyopia is a monocular or binocular visual dysfunction caused by form deprivation and abnormal binocular interaction during the critical period of the visual cortex. It is one of the most common causes of unilateral vision impairment in children and young adults, and the incidence is reported to range 1%–3.5% (1, 2). Amblyopia is thought to be related to dysfunction in the processing of visual information or a deficit in optotype acuity in the absence of ocular and neural pathological changes (3, 4). The risk factors of amblyopia include anisometropia, strabismus, form deprivation, and high bilateral refractive errors (5). Hyperopic anisometropia is the most frequent risk factor for amblyopia. Amblyopic eyes are structurally normal on clinical examinations.

In recent years, whether the microscopic retina and choroid are changed in the patients with amblyopia has aroused widespread attention. The choroid plays a role in the growth of sclera, hence regulating emmetropization (6). Choroid has been shown to be involved in the development of refractive state and axial elongation in animal models (7). Hung et al. (8) found changes in the monkey eye's effective refractive state produce rapid compensating changes in choroidal thickness (CT), and the choroidal changes may play an important role in the visual regulation of axial growth associated with emmetropization. In addition to animal experiments, many current studies have confirmed that the choroidal structures in anisometropic amblyopic eyes of children were significantly different from that of normal eyes. A meta-analysis of unilateral amblyopia reported that CT increased in amblyopic eyes (9). Although recent studies have shown that amblyopic eyes exhibit greater sub-foveal CT than that of their fellow eyes and age-matched control eyes (10–13), it is also quite controversial whether the choroidal microstructural changes are involved in the pathogenesis of amblyopia (11).

Since various variables can affect choroidal thickness in children, such as age, spherical equivalent, and axial length (AL), it is necessary to explore more reliable marker for the assessment of choroidal vascular structural characteristics. Agrawal et al. (14) proposed a new parameter-choroidal vascularity index (CVI) to quantitative assess choroidal vascular structure by calculating the ratio of vascular luminal area (LA) to total choroidal total choroidal area (TCA) through enhanced depth imaging optic coherence tomography (EDI-OCT) images. With growing evidence, CVI is emerging as a potentially more robust marker for choroidal vascularity in various ocular diseases (14–16). Nishi et al. (17) reported a higher choroidal luminal/stromal ratio in amblyopic eyes than in fellow and control eyes, implicating morphological differences in the choroid may be a factor in amblyopia. Baek et al. (18) found choroidal vascularity was higher in amblyopic eyes. Although previous studies have evaluated CT changes in amblyopia eyes, the relationship between amblyopia and choroidal blood perfusion has not yet been adequately studied, and there is no consensus about whether the amblyopic choroid vascularity is structurally abnormal in children. The aim of this study was to assess the choroidal microstructural and choroidal blood perfusion changes and assess the correlation between AL, CT, and choroid vascular microstructure parameters in preschool-age children with anisometropic hyperopic amblyopia.

## Materials and methods

### Subjects

This was a cross-sectional comparative, non-interventional study conducted at Beijing Chaoyang Hospital, Capital Medical University from January 2022 to July 2022. This study was conducted in accordance with the tenets of the World Medical Association's Declaration of Helsinki and approved by the ethics committee of Beijing Chaoyang Hospital, Capital Medical University. The parents were also informed about the study and procedures, and written informed consent was obtained from all the patients and controls or their parents to perform the measurements and to review their medical records.

This study involved 52 hyperopic anisometropic amblyopia children and 48 age-matched heathy controls. The inclusion criteria were as follows: age between 3 and 10 years; IOP lower than 21 mmHg; normal anterior chamber angles; normal optic nerve head without glaucomatous changes, such as the neuroretinal rim narrowing, cup-disc ratio increasing; and no retinal nerve fiber layer abnormalities. Patients with a history of ocular or systemic diseases, including strabismus, organic eye diseases, history of intraocular surgery, cataract, neurologic disease, glaucoma, or any other retinal disorders; Spectralis OCT images with a quality score less than 20 or erroneous segmentation, illumination, or centration were excluded.

### Ophthalmic examination

All the patients and controls had dilated funduscopic examinations. Best-corrected vision acuity (BCVA), slit-lamp biomicroscopy, AL (IOL Master; Carl Zeiss Meditec, Dublin, CA), spherical equivalent (SE) and intraocular pressure (IOP) were measured. The visual acuity was measured with a standard logarithmic visual acuity chart, and the decimal visual acuity was converted to logMAR units for the statistical analyses.

OCT imaging of the optic nerve head (ONH), the retinal nerve fiber layer (RNFL), and choroidal architectural parameters were obtained using the Spectralis OCT device (Heidelberg Engineering, Heidelberg, Germany) by experienced ophthalmologist. The peripapillary RNFL thickness (pRNFLT) was measured through the dilated pupil using OCT, scans were centered on the optic disc, and the 12° scan circle was positioned exactly in the middle of the optic nerve head. Utilizing the Fast RNFL program, the RNFLT was determined around a set diameter (3.5 mm) from the center of the optic disc. The global (G), nasal (N), nasal superior (NS), nasal inferior (NI), temporal (T), temporal superior (TS) and temporal inferior (TI) RNFLT measurements were recorded and analyzed.

**Figure 1:**
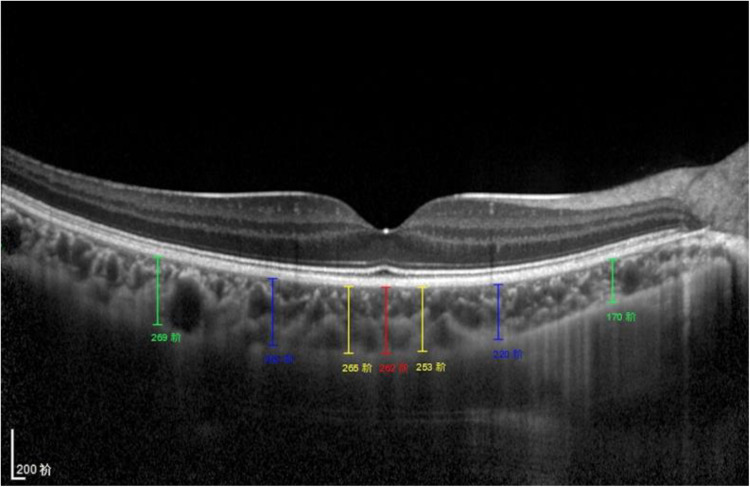
Subfoveal CT measurement. CT was measured at 7 points: directly beneath the fovea or the subfoveal area (SFCT) point, and 0.5/1.5/3 mm to the fovea nasally (N1/N2/N3), 0.5/1.5/3 mm to the fovea temporally (T1/T2/T3).

**Figure 2:**
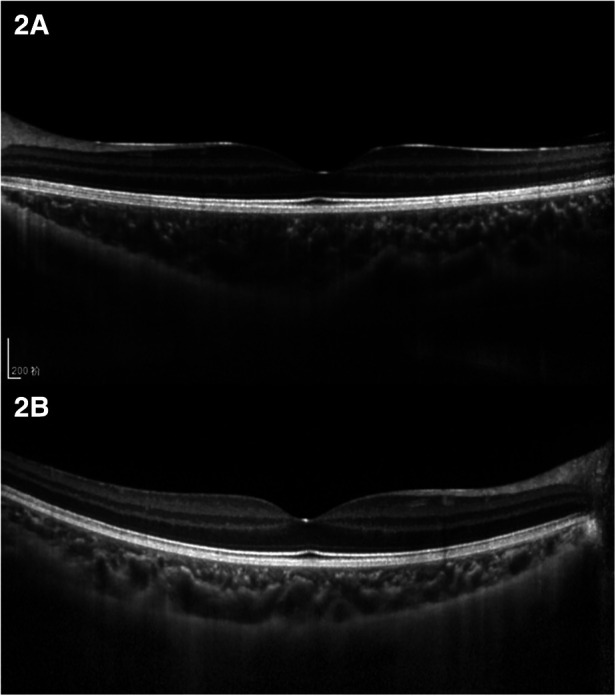
Comparison of choroidal structure of macula in a patient with anisometropia amblyopia. (**A**) Choroidal thickness of the hyperopic anisometropic amblyopia eye. (**B**) Choroidal thickness of the fellow eye.

**Figure 3:**
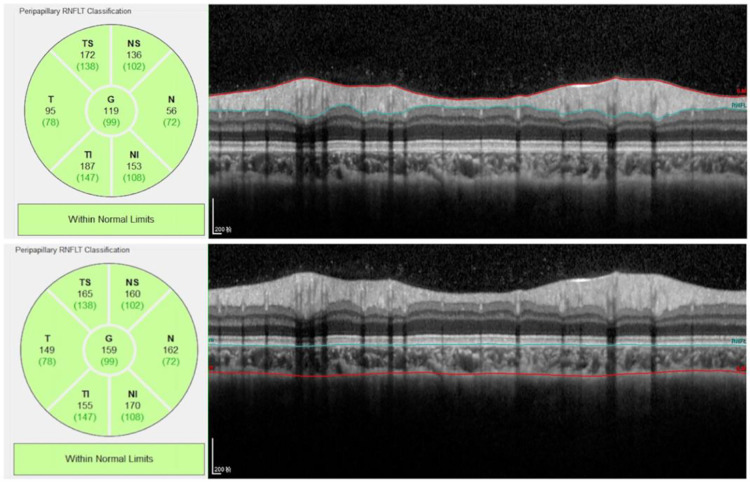
Peripapillary RNFL and choroidal thickness measurement. The RNFL thickness data can be automatically calculated and displayed. For peripapillary choroidal thickness measurement, we manually moved the automatically segmented internal limiting membrane line to the choroid sclera junction and moved RNFL line to the retina pigment epithelial line. Once we changed the automatically segmented line, the choroidal thickness data were automatically calculated and displayed.

The choroid was imaged using the EDI mode of OCT. The macular region was scanned using a 7 horizontal line scan centered on the fovea, with 100 frames averaged in each B-scan. For the macular CT measurement, 7 points were selected for manual measurement: the subfoveal CT (SFCT) point, the temporal and nasal points at a radius of 0.5-mm, 1.5-mm, and 3-mm (Figure 1). The comparison of macula choroidal structure in a patient with anisometropia amblyopia was shown in Figure 2. For peripapillary CT (pCT) measurement, a 3.5 mm diameter area centered on optic disc was selected for measurement. The pRNFLT data can be automatically calculated and displayed. For pCT measurement, we manually moved the automatically segmented internal limiting membrane line to the choroid sclera junction and moved RNFL line to the retina pigment epithelial line. Once we changed the automatically segmented line, the pCT data were automatically calculated and displayed (Figure 3). Three consecutive measurements were performed by one experienced ophthalmologist and the average of three measurements was used for statistical analyses. All analyses were corrected for the magnification effect. Littmann formula was used to calculate true image size, as described previously (19, 20).

**Figure 4:**
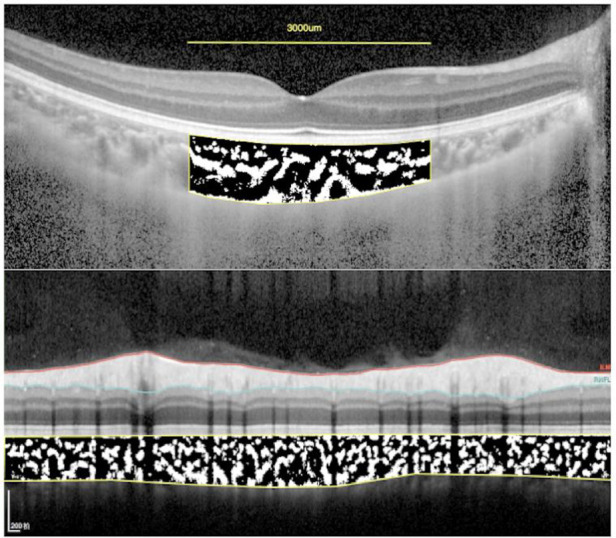
The subfoveal choroidal area and peripapillary choroidal area measurement. Using ImageJ software, the image was binarized with Niblack's method, and the ratio of vascular area (black pixels) to stromal area (white pixels) was quantified.

OCT system uses fixed eye AL to scan. With the elongation of AL, the scanning area increases, resulting in optical amplification effect. Measurements performed with OCT have inherent errors when the scale of retinal image is not corrected for AL of each eye (20, 21). In order to correct the optical amplification effect of OCT measurement, Littmann formula was used to calculate true image size make the results more accurate. The measured OCT image diameter (*D*_m_) and the true diameter (*D*_t_) can be converted by Littmann formula: $D_{t}=p\times q\times D_{m}$, *p* is the magnification factor of the imaging system and *q* is a factor related to the eye [q=0.01306×(AL−1.82)], *p* is a constant 3.46 according to the previous study (22).


$$D_{t}=3.46\times 0.01306\times \left(AL-1.82\right)\times D_{m}$$

### Binarization of EDI OCT images

For the measurement of sub-fovea choroidal area, a 3,000-μm wide area was chosen. For the measurement of peripapillary choroidal area, the total circumferential choroid area with a diameter of 3.5 mm centered at the fovea was chosen to determine the binarization (Figure 4). The choroidal vascular structure parameters were measured by Niblack method using ImageJ software version 1.47 (National Institutes of Health, Bethesda, MD, United States) as described previously (23). In brief, Niblack's auto local threshold tool was applied to allow demarcation of LA, the stromal area (SA), and TCA. The proportion of LA to TCA was defined as CVI.

### Statistical analysis

The Kolmogorov–Smirnov test was used to identify the normality of distribution. Descriptive statistics and optic characteristics were calculated as the mean and standard deviation for normally distributed variables. Age differences were performed with the Independent-samples T test. The categorical data were analyzed using the Fisher's exact test. One-way analysis of variance was used to compare the differences among the three groups, and the Bonferroni method was used for *post hoc* tests. The significance of the correlations between the ocular parameters and choroid microstructure parameters was determined by Pearson's correlation coefficient. All reported *P* values were two sided. A *P*-value < 0.05 was regarded as statistically significant. Statistical analysis was performed using the SPSS software version 26 (SPSS, Inc., IL, United States).

## Results

### Demographic data and clinical characteristics

The demographic and ocular characteristics were analyzed in the study (Table 1). The mean age of the subjects with amblyopia was 5.65 ± 1.22 years (range 3 to 9 years), and the mean age in the control group was 5.71 ± 0.80 years (range 4 to 9 years). No significant difference between the mean age of the groups was observed.

**Table 1:** Demographic and ocular characteristics.

Parameters	Unilateral amblyopic children		Controls	*P*1	*P*2	*P*3
	Amblyopic eyes	Fellow eyes				
No. eyes (patients)	52		48	–	–	–
Age, years, mean ± SD	5.65 ± 1.22		5.71 ± 0.80	–	–	*P* = 0.513*
Male, *n* (%)	24 (46.15%)		22 (45.83%)	–	–	*P* = 0.615^†^
SE, D, mean ± SD	6.16 ± 1.54	3.84 ± 1.99	0.31 ± 0.43	***P* < 0.001** ^‡^	***P* < 0.001**	***P* < 0.001**
BCVA	0.46 ± 0.22	0.10 ± 0.18	−0.01 ± 0.07	***P* < 0.001** ^‡^	***P* < 0.001**	***P* < 0.001**
AL (mm)	21.02 ± 1.07	21.96 ± 1.29	23.24 ± 0.54	***P* < 0.001** ^‡^	***P* < 0.001**	***P* < 0.001**

AL, axial length; BCVA, best corrected visual acuity; SE, spherical equivalent; SD, standard deviation; D, diopter; logMAR, minimal angle of logarithm.

Data are expressed as the means ± standard deviations.

*P*1, Significant difference among amblyopic eyes, fellow eyes, and controls.

*P*2, Significant difference between amblyopic eyes and fellow eyes.

*P*3, Significant difference amblyopic eyes and controls.

* Independent-samples *T* test.

^†^ Chi-square test.

^‡^ One-way ANOVA test, all comparisons were corrected with *post hoc* test.

The mean BCVA was 0.46 ± 0.22 logMAR units in the amblyopic eyes, 0.10 ± 0.18 logMAR units in the fellow eyes, and −0.01 ± 0.07 logMAR units in the controls. The mean BCVA was significantly worse in the amblyopic eyes than the other two groups (*P* < 0.001). The mean SE of amblyopic eyes, fellow eyes and controls were 6.16 ± 1.54 D (range +3.00 to +10.00 D), 3.84 ± 1.99 D (range 0.50 to + 8.0 D) and 0.31 ± 0.43 D (range 0.38 to +1.25 D), respectively. The mean AL was significantly lower in the amblyopic eyes than in other groups.

### Macular choroidal parameters in amblyopic eyes, fellow eyes, and controls

The mean SFCT was 323.39 ± 35.37 μm, 290.07 ± 48.04 μm, and 278.40 ± 24.10 μm in the amblyopic eyes, fellow eyes, and controls, respectively (Table 2). The subfoveal choroid of the amblyopic eyes was significantly thicker than that of the fellow eyes and control eyes (*P* < 0.001). The nasal choroidal sectors at 1.5 mm and 3.0 mm diameter of the amblyopic eyes were thicker than that of the fellow eyes and control eyes (*P* < 0.001). The choroidal thickness at 1.5 mm and 3.0 mm diameter was not significantly different among the three groups (*P* > 0.05).

**Table 2:** Macular choroidal parameters in amblyopic eyes, fellow eyes, and controls.

Choroidal parameters	Unilateral amblyopic children		Controls	*P*1	*P*2	*P*3
	Amblyopic eyes	Fellow eyes				
**CT, μm, mean ± SD**						
SFCT	323.39 ± 35.37	290.07 ± 48.04	278.40 ± 24.10	***P* < 0.001**	***P* < 0.001**	***P* < 0.001**
Nasal 1.5 mm	289.22 ± 30.49	254.77 ± 46.84	231.46 ± 38.66	***P* < 0.001**	***P* < 0.001**	***P* < 0.001**
Nasal 3 mm	216.88 ± 39.96	204.65 ± 42.78	157.32 ± 27.29	***P* < 0.001**	*P* = 0.084	***P* < 0.001**
Temporal 1.5 mm	299.41 ± 38.95	290.79 ± 40.81	300.40 ± 31.36	*P* = 0.301	*P* = 0.212	*P* = 0.848
Temporal 3 mm	258.90 ± 41.32	260.75 ± 35.70	272.60 ± 40.60	*P* = 0.157	*P* = 0.848	*P* = 0.078
**Choroidal Vascular Parameters**						
LA, μm^2^, mean ± SD	1,555,572.28 ± 240,531.00	1,434,537.31 ± 317,146.02	1,392,546.06 ± 183,442.60	***P* = 0.005**	***P* = 0.017**	***P* = 0.002**
SA, μm^2^, mean ± SD	915,837.31 ± 143,148.45	790,779.50 ± 216,348.44	758,347.15 ± 79,039.03	***P* < 0.001**	***P* < 0.001**	***P* < 0.001**
TCA, μm^2^, mean ± SD	2,471,409.59 ± 318,427.55	2,225,316.81 ± 343,679.17	2,150,893.20 ± 225,859.85	***P* < 0.001**	***P* < 0.001**	***P* < 0.001**
CVI	0.63 ± 0.04	0.64 ± 0.09	0.65 ± 0.03	***P* = 0.018**	*P* = 0.244	***P* = 0.041**

CT, choroidal thickness; CVI, choroidal vascularity index; SFCT, subfoveal choroidal thickness; SA, stromal area; LA, luminal area; TCA, total choroidal area.

One-way ANOVA test, all comparisons were corrected with *post hoc* test.

*P*1, Significant difference among amblyopic eyes, fellow eyes, and controls.

*P*2, Significant difference between amblyopic eyes and fellow eyes.

*P*3, Significant difference amblyopic eyes and controls.

For the choroidal vascular parameters, SA values were 915,837.31 ± 143,148.45 μm^2^ in the amblyopic eyes, 790,779.50 ± 216,348.44 μm^2^ in the fellow eyes, 758,347.15 ± 79,039.03 μm^2^ in the control eyes; LA values were 1,555,572.28 ± 240,531.00 μm^2^, 1,434,537.31 ± 317,146.02 μm^2^, and 1,392,546.06 ± 183,442.60 μm^2^ among the three groups, respectively. TCA values were 2,471,409.59 ± 318,427.55 μm^2^ in the amblyopic eyes, 2,225,316.81 ± 343,679.17 μm^2^ in the fellow eyes, 2,150,893.20 ± 225,859.85 μm^2^ in the control eyes; SA, LA and TCA of the amblyopic eyes were significantly larger than that of the fellow and control eyes (all *P* < 0.05). The CVI values of the amblyopic eyes was significantly different among the three groups (*P* = 0.018).

**Figure 5:**
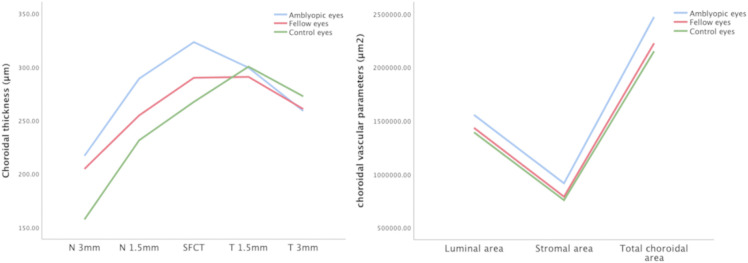
Choroidal thickness and vascular parameters among the three groups. The subfoveal choroidal thickness, temporal and nasal choroidal thickness, LA, SA, and TCA values of amblyopic eyes, fellow eyes, and controls.

CT and choroidal vascular parameters among the three groups were shown in Figure 5.

### Peripapillary RNFL thickness and choroidal parameters

The mean global and temporal superior RNFLT in the amblyopic eyes were significantly higher than those of normal control eyes (all *P* < 0.05; Table 3). The pCT in all directions of the amblyopic eyes was significantly thicker than the control eyes (all *P* < 0.05).

**Table 3:** Peripapillary RNFL thickness and choroidal parameters in the three groups.

Parameters	Unilateral amblyopic children		Controls	*P*1	*P*2	*P*3
	Amblyopic eyes	Fellow eyes				
RNFL, μm, mean ± SD						
G	97.37 ± 9.56	94.51 ± 5.83	92.37 ± 6.98	***P* = 0.005**	*P* = 0.058	***P* = 0.001**
T	74.92 ± 7.13	72.21 ± 8.90	72.60 ± 6.36	*P* = 0.148	*P* = 0.070	*P* = 0.127
TS	132.28 ± 11.94	127.71 ± 13.95	123.80 ± 8.73	***P* = 0.002**	*P* = 0.050	***P* < 0.001**
TI	141.09 ± 12.97	139.83 ± 14.18	136.99 ± 22.16	*P* = 0.463	*P* = 0.702	*P* = 0.224
N	69.47 ± 15.60	68.47 ± 11.08	64.77 ± 10.22	*P* = 0.152	*P* = 0.687	*P* = 0.064
NS	116.06 ± 15.44	115.46 ± 16.73	114.79 ± 19.30	*P* = 0.934	*P* = 0.859	*P* = 0.712
NI	114.16 ± 13.74	112.38 ± 16.78	114.62 ± 19.87	*P* = 0.780	*P* = 0.592	*P* = 0.893
**CT, μm, mean ± SD**						
G	156.62 ± 20.21	150.74 ± 25.52	143.62 ± 12.57	***P* = 0.007**	*P* = 0.142	***P* = 0.002**
T	161.99 ± 26.72	148.31 ± 22.78	144.88 ± 11.14	***P* = 0.001**	***P* = 0.004**	***P* < 0.001**
TS	160.18 ± 21.20	157.32 ± 28.15	147.65 ± 9.75	***P* = 0.011**	*P* = 0.495	***P* = 0.004**
TI	151.00 ± 22.93	145.10 ± 28.50	138.52 ± 11.11	***P* = 0.022**	*P* = 0.179	***P* = 0.006**
N	151.35 ± 27.58	147.58 ± 31.97	137.58 ± 13.19	***P* = 0.025**	*P* = 0.458	***P* = 0.008**
NS	156.79 ± 27.39	155.22 ± 33.38	139.63 ± 12.68	***P* = 0.002**	*P* = 0.773	***P* = 0.004**
NI	151.45 ± 26.84	144.65 ± 16.55	135.37 ± 16.56	***P* = 0.011**	*P* = 0.191	***P* = 0.003**
**Choroidal Vascular Parameters**						
SA	2,897,700.89 ± 471,994.36	2,483,938.16 ± 380,585.43	2,459,067.96 ± 241,667.84	***P* < 0.001**	***P* < 0.001**	***P* < 0.001**
LA	5,011,039.04 ± 719,632.91	4,481,049.92 ± 737,795.37	4,596,272.08 ± 548,053.75	***P* < 0.001**	***P* < 0.001**	***P* = 0.003**
TCA	7,908,739.94 ± 962,558.03	6,964,988.08 ± 1,092,260.95	7,055,340.04 ± 711,275.62	***P* < 0.001**	***P* < 0.001**	***P* < 0.001**
CVI	0.63 ± 0.04	0.64 ± 0.02	0.65 ± 0.02	***P* = 0.017**	*P* = 0.110	***P* = 0.005**

CT, choroidal thickness; G, global; N, nasal; NS, nasal superior; NI, nasal inferior; RNFL, retinal nerve fiber layer; T, temporal; TS, temporal superior; TI, temporal inferior; SA, stromal area; LA, luminal area; TCA, total choroidal area; CVI, choroidal vascularity index.

One-way ANOVA test, all comparisons were corrected with *post hoc* test.

*P*1, Significant difference among amblyopic eyes, fellow eyes, and controls.

*P*2, Significant difference between amblyopic eyes and fellow eyes.

*P*3, Significant difference amblyopic eyes and controls.

For the peripapillary choroidal vascular parameters, SA values were 2,897,700.89 ± 471,994.36 μm^2^ in the amblyopic eyes, 2,483,938.16 ± 380,585.43 μm^2^ in the fellow eyes, 2,459,067.96 ± 241,667.84 μm^2^ in the control eyes; LA values were 5,011,039.04 ± 719,632.91 μm^2^, 4,481,049.92 ± 737,795.37 μm^2^, and 4,596,272.08 ± 548,053.75 μm^2^ among the three groups, respectively. TCA values were 7,908,739.94 ± 962,558.03 μm^2^ in the amblyopic eyes, 6,964,988.08 ± 1,092,260.95 μm^2^ in the fellow eyes, 7,055,340.04 ± 711,275.62 μm^2^ in the control eyes; SA, LA and TCA of the amblyopic eyes were significantly larger than that of the fellow eyes and control eyes (all *P* < 0.001). There was significant statistical difference of the CVI values among the three groups (*P* = 0.017).

### Correlation analyses between the parameters and choroid microstructure parameters

A correlation analysis between parameters and choroid microstructure parameters were shown in Table 4. There was a statistically significant negative correlation between AL and SFCT, LA and TCA levels (*r* = −0.372, −0.193 and −0.280, respectively; *P* < 0.001, *P* = 0.039, *P* = 0.027, respectively). There was a statistically significant positive correlation between SE, SFCT, LA and TCA levels (*r* = 0.456, 0.229 and 0.240, respectively; *P* < 0.001, *P* = 0.019, 0.014, respectively). SFCT was positive correlated with LA, SA, TCA and CVI levels (r = 0.557, 0.197, 0.561 and 0.231, respectively; all *P* < 0.05).

**Table 4:** Correlation analyses between the parameters and choroid microstructure parameters.

Parameters	SFCT		LA		SA		TCA		CVI	
	*r*	*P*	*r*	*P*	*r*	*P*	*r*	*P*	*r*	*P*
**Age**	0.019	0.847	−0.016	0.874	0.163	0.098	0.077	0.437	−0.146	0.139
**AL**	−0.372	**<0**.**001**	−0.193	**0**.**039**	−0.071	0.472	−0.280	**0**.**027**	−0.084	0.398
**SE**	0.456	**<0**.**001**	0.229	**0**.**019**	0.098	0.324	0.240	**0**.**014**	0.086	0.385
**Choroid parameters**										
SFCT	-	-	0.557	**<0**.**001**	0.197	**0**.**046**	0.561	**<0**.**001**	0.231	**0**.**018**
Nasal 3 mm	0.291	**0**.**003**	0.306	**0**.**002**	0.285	**0**.**003**	0.406	**<0**.**001**	−0.053	0.593
Temporal 3 mm	0.214	**0**.**029**	0.232	**0**.**018**	0.254	**0**.**009**	0.328	**0**.**001**	−0.070	0.479

AL, axial length; SFCT, subfoveal choroidal thickness; SE, Spherical equivalent; SA, stromal area; LA, luminal area; TCA, total choroidal area; CVI, choroidal vascularity index.

## Discussion

In the present study, we determine the pRNFLT, SFCT, pCT, and choroid microstructure parameters in children with hyperopic anisometropic amblyopia and compare that of fellow eyes and age-matched controls. We also analyze the correlation between the AL, CT, and choroid microstructure parameters.

The results of different studies on the changes of nerve fiber layer thickness in hyperopia patients are controversial. Most studies suggested that the pRNFL of children with high hyperopia was significantly thicker than low hyperopia or emmetropic children (24–26). While Qian et al. (27) found that the RNFL in the inferior and temporal quadrants in the 1 to 3 mm diameter of the macular fovea in moderate-to-high hyperopia children were thinner than those in emmetropic children. In the present study, the mean global and temporal pRNFLT were significantly higher than those of normal control eyes. To date, it is not clear why the RNFLT in hyperopia children is changed. This may be due to the influence of retinal development in children with hyperopia. There are also several studies explored the factors associated with RNFL in different types of ametropia patients. It is suggested that SE or AL was associated with RNFL thickness (28–30). Eslami et al. (31) found RNFLT in Iranian children aged below 18 years positively correlated with SE, but no significant association between RNFLT and age. In our study, we also found that highly hyperopic children with a shorter eye axis had thicker pRNFLT than the normal controls.

We also analyzed the macular and peripapillary choroid microstructure parameters in hyperopic anisometropic amblyopia eyes. The choroid plays a role in refractive error development and has been shown to be involved in the visual feedback pathway in humans (6). The question of whether the choroid is affected in human amblyopia is an important issue for ophthalmologists. Previous studies showed that the subfoveal choroid of hyperopic amblyopia eyes was significantly thicker than that of the fellow and the control eyes (9, 11, 12, 13, 32–36). These results suggesting that amblyopia might have a profound influence on CT (32). In the present study, we found SFCT and pCT in all directions of the amblyopic eye was significantly thicker than the fellow and control eyes. CT was influenced by age, sex, AL, or refraction (37–39). Recently different researchers had different hypotheses on the mechanisms of choroidal thickness increased in anisometropic hyperopic amblyopia eyes. Bidaut-Garnier et al. (40) and Nagasawa et al. (38) reported that CT was negatively correlated with AL in healthy people. Mori et al. (36) found a negative correlation between the CT and AL in preschool children with hyperopic anisometropic. However, the causal relationship between choroidal thickening and hyperopia remains controversial. Nishi and his colleagues described the possibility that the increased SFCT of amblyopic eyes is under the influence of the amblyopia (13). Kara et al. (41) thought that amblyopia affected the development process of the choroids. Troilo et al. (42) believe that the thickened choroid might hinder the growth of the eye during development, it provides a barrier to the diffusion of growth factors or acts as a mechanical buffer to limit the eye's elongation. Another idea is that the foveola was thicker in the amblyopic eyes, and a thicker retina may require additional blood for nourishment, then the CT increased to supply additional blood. At the same time, it is also believed that the thickening of choroid will affect the axis length elongation. It is reported that the choroid thickens during the normal development of primate eyes, which may slow down the growth of the eye during development (42).

Myopic or hyperopic defocus may also affect choroid thickness. The choroid becomes thicker with myopic defocus and thinner with hyperopic defocus, which in turn adjusts the position of the retina to maintain clear vision (9). Hung et al. (8), and Nishi et al. (13) suggested hyperopic defocus promotes choroidal thinning, myopic defocus promotes choroidal thickening. Nishi et al. (13) also hypothesized that the ocular compensation and choroidal accommodation for the hyperopic defocus was suppressed in amblyopic eyes, which resulted in an increased SFCT. These reports suggested that modulation of choroidal thickness is a response to optical defocus. Myopic defocus induces choroidal thickening and develops hyperopia, while hyperopic defocus induces choroidal thinning and leads to myopia. The ocular growth with emmetropization after birth is regulated by visual feedback from visual signals on the retina, effective visual stimuli and visual feedback mechanism may also affect choroid microstructure changes. Previous animal experiment reports raised the possibility that choroid is involved in the visual feedback (43, 44). Mori et al. (36) concluded that choroidal accommodation may be inhibited with the little visual feedback in the amblyopic eye in hyperopic anisometropic amblyopia, and the anomalous subfoveal choroidal thicknesses may reflect a delay in emmetropization. Troilo et al. (42) found that the choroid thickens during postnatal eye growth, which may slow down the eye growth during development. However, it is not clear whether the lack of visual stimulation in patients with high hyperopia causes the thickening of the choroid, or the thickening of the choroid causes the restriction of axial elongation, which leads to the occurrence of hyperopia.

Most studies suggesting that the choroid is thicker in amblyopic eyes than in normal eyes, but CT can be affected by various variables. The choroid is composed of abundant blood vessels surrounded by stromal tissue, the role of choroid tissue in amblyopia may be related to the blood supply to the outer retina, choroidal thickness only cannot fully reflect the choroid structural changes and blood supply situation. Agrawal et al. (14) proposed CVI to assess choroidal vascular structure by calculating the LA to TCA ratio through EDI-OCT images. In recent years, these parameters had been used widely used in the field of refractive error research.

Ruiz-Medrano (45) reported that the average percentage of the vascularity was 60.56% in children. However, there are conflicting views on choroidal blood perfusion in the pediatric hyperopia research field. Baek et al. claimed that the choroidal CV was higher in both hyperopia and the fellow eyes compared to normal eyes, but no significant difference was found between amblyopic eyes and fellow eyes choroidal LA to TCA ratio (i.e., CVD) was higher in hyperopia eyes than the control eyes; Eraydın et al. reported that they did not find any difference in CVI in hyperopic amblyopic eyes compared with the control group (18, 46). Enrico Borrelli et al. (47) reported that amblyopic eyes were found to have increased choriocapillaris vessel density and speculated an increase in choriocapillaris vessel density might be a compensatory response that supplies more blood to a thickened outer retina in amblyopic eyes. But most studies concluded that the choroidal blood flow in patients with high hyperopia is lower than that in normal children. Huang et al. (48) found the choriocapillaris flow void (FV) in the amblyopic group was greater than that in the age- and sex-matched heathy control group and concluded that children with amblyopic eyes have attenuated macular and choriocapillaris perfusion. There are other studies reported that both the amblyopic and the fellow eyes also had lower CVI values than control eyes (49–51). The results in this study are consistent with most of the previous studies, the average CVI in the hyperopic amblyopic eyes was lower than the fellow eyes and the normal control eyes. When exploring the relationship between choroidal vascularity parameters and choroid thickness, different researchers have drawn opposite conclusions. Beak et al. (18) reported the choroidal vascularity was negatively correlated with CT may suggests insufficient blood supply to the outer retina and choroid in the affected eyes of patients with unilateral anisometropic hyperopic amblyopia. Fujiwara et al. (52) found SCT had a significant positive relationship with vascular density of the choroid in normal eyes. In our cohort, the average CVI of sub-fovea choroidal area was comparable at 0.63 ± 0.04 in the amblyopic eyes, 0.64 ± 0.09 in the fellow eyes and 0.65 ± 0.03 in the control eyes (*P* = 0.002), and SCT had a slight negative correlation with CVI, but the difference was not statistically significant. Studies on CVI changes in amblyopia patients have shown controversial results, which may be due to the following reasons. First, the age of the enrolled patients in different studies varies, which may affect the conclusion, because age may affect choroidal thickness and choroidal blood flow. The second possible reason is that current studies have shown that amblyopia treatment may affect the choroid structure changes. Nishi et al. (53) showed that the amblyopic eyes had a larger LA than control eyes at the baseline, but wearing the optical correction led a significant decrease in the LA and widened SA in the amblyopic eyes one year after the treatment. Thirdly, differences in results among these studies may thus be due to differences in the methods used for choroidal blood flow analysis. Some studies use binary OCT images to analyze the macular choroidal fault surface. However, other studies used OCTA scanning for automatic blood flow analysis. The choroid position and section selected by these two measurement methods are different, so there may be differences in research results.

At present, it is controversial whether the thickening of choroid in hyperopia is caused by the increase of stromal area or the vascular luminal area. Ruiz-Medrano et al. (45) reported that the LA and the percentage of vascular/total area decreased with increasing age in normal children, while SA remains stable. Alis et al. (54) found that TCA and SA were higher in hyperopia than in both emmetropic and myopic eyes, they also proposed that the reason for the decrease in the CVI in hyperopia is the excess of the SA. Nishi et al. hold different opinions that the LA was significantly larger, the SA was significantly smaller, and the luminal/stromal ratio was larger in amblyopic eyes than in control hyperopic eyes (17). Kaderli et al. (55) found both refractive error increase and axial length decrease have resulted in an increase in largest macular choroidal vascular lumen diameters and areas in hyperopic adults. Araki showed that choroidal blood flow with respect to total choroidal volume may be increased in amblyopic eyes than in fellow and normal control eyes (33). Terada et al. (56) found that the outer choroidal vascular area in both amblyopic and fellow eyes was markedly larger than in healthy eyes, and they also speculated that an outer choroidal vascular area >59% in fellow eyes with normal vision, may indicate a risk for amblyopia onset. This finding might be helpful in detecting amblyopia risk before onset in many young-age children (56). As to why the choroidal vascular area in anisometropic hyperopic amblyopia eyes increases, some researchers have also conducted research. Guo et al. (57) discovered that most amblyopic eyes displayed a dark atrophic patch, the choroidocapillary atrophy patch caused a wide range of compensatory dilatation of its surrounding capillaries, and dilatation of the choroidal vessels may lead to increased CT. This study showed that the SA, LA, and TCA in the amblyopic eyes was significantly larger than that of the fellow and the control eyes, the larger LA and SA at the baseline was characteristic of the amblyopic eye. The results indicate that choroidal blood flow with respect to total choroidal volume may be increased in amblyopic eyes than in fellow and normal control eyes.

Some studies further explored the choroid structure changes before and after amblyopia treatment. LA was characteristic of the amblyopic eye, and it was significantly reduced after the optical treatment. Nishi et al. (53) showed that the amblyopic eyes had a larger LA than control eyes at the baseline, but wearing the optical correction led a significant decrease in the LA and widened SA in the amblyopic eyes one year after the treatment. Toor et al. (58) suggested that the amblyopic eyes with widened SA had more nonvascular smooth muscle cells and had better accommodation, which induced the improvement of the visual acuity. Thus, the widened SA in the amblyopic eyes after treatment is probably a response to the optical correction of the refractive error. The amblyopic eyes with larger SA at the baseline had better improvements of the visual acuity.

The correlation between the AL, CT and choroid microstructure parameters were also evaluated in the present study, there was a statistically significant negative correlation between AL and SFCT, LA and TCA levels, and a positive correlation between SE, SFCT and LA levels. Kaderli et al. (55) found macular CT increased with increasing hyperopic refractive error and decreasing AL in all quadrants. Nishi et al. (17) showed that there was a significant negative correlation between the LA/SA ratio and AL in the control eyes, but no significant correlation was found in the amblyopic eyes. Baek et al. (18) found a negative correlation between subfoveal CT and choroidal vascularity in amblyopic eyes. Araki et al. (33) reported the SFCT was significantly positive associated with CVD. Ruiz-Medrano et al. (45) reported that the LA and the percentage of LA/TCA decreased with increasing age while the SA remained stable in normal children and adults.

The present study had some limitations. First, the sample size was small. A further study of a larger number of subjects will be necessary to confirm our findings. Secondly, we did not discuss the correlation between choroid structural and blood flow changes with age, gender, and other factors, so we will expand the sample size in the further research to explore this. Third, the measurement of CVI may be difficult, as it requires the acquisition of good quality EDI-OCT scans, the binarization of the image will be inaccurate if the fundus picture is not clear enough, poor-quality OCT images or eye movement further limit the detailed choroidal evaluation and CVI calculation. In addition, in the process of image grading, there is a step that requires doctors to judge the choroidal boundary, which requires high requirements for the operator.

## Conclusions

In conclusion, the current study showed that the choroidal structure of the amblyopic eyes was different from the fellow and the normal control eyes, the hyperopic anisometropic amblyopic eyes had significantly thicker sub-foveal choroid, higher LA, SA, and TCA. The subfoveal and peripapillary choroidal thickness of amblyopic children abnormally increased and the thicker subfoveal choroid is mildly correlated with their shorter axial length. AL and CT affect choroidal structure and vascular density of the choroid. Choroidal blood flow may be decreased in amblyopic eyes. The larger LA, SA, TCA, and lower CVI were characteristic of the amblyopic eye.

## Data Availability

The raw data supporting the conclusions of this article will be made available by the authors, without undue reservation.
